# Immediate implants following tooth extraction. A systematic review

**DOI:** 10.4317/medoral.17469

**Published:** 2011-12-06

**Authors:** Jordi Ortega-Martínez, Tania Pérez-Pascual, Santiago Mareque-Bueno, Federico Hernández-Alfaro, Eduard Ferrés-Padró

**Affiliations:** 1 DDS, Master in Oral Implantology, PhD Student, Medicine, Surgery and Oral Implantology Department, Dental School, International University of Catalonia; 2 DDS, Master in Oral Implantology, PhD Student, Medicine, Surgery and Oral Implantology Department, Dental School, International University of Catalonia; 3 DDS, Master in Oral Implantology, PhD Student, Medicine, Surgery and Oral Implantology Department, Dental School, International University of Catalonia; 4 MD, DDS, PhD OMFS, Prof. And Director Master in Oral Implantology, Medicine, Surgery and Oral Implantology Department, Dental School, International University of Catalonia; 5 MD, DDS, PhD OMFS, Prof. And Head of Department of Medicine, Surgery and Oral Implantology Department, Dental School, International University of Catalonia

## Abstract

Objectives: The aim of this article is to review the current state of immediate implants, with their pros and contras, and the clinical indications and contraindications. Material and Methods: An exhaustive literature search has been carried out in the COCHRANE library and MEDLINE electronic databases from 2004 to November 2009. Randomized clinical trials and clinical trials focused on single implants placed in fresh extraction sockets were included and compared. A meta-analysis could not be performed due to heterogeneity of the data. Results: Twenty studies out of 135 articles from the initial search were finally included, which summed up a total of 1139 immediate implants with at least a 12-month follow-up. Our results have been compared with other current available papers in the literature reviewed that obtained similar outcomes. Discussion: Immediate implants have predictable results with several advantages over delayed implant placement. However, technical complications have been described regarding this technique. Also, biomaterials may be needed when the jumping distance is greater than 1mm or any bone defect is present. Conclusions: Few studies report on success rates rather than survival rates in the literature reviewed. Short-term clinical results were described and results were comparable to those obtained with delayed implant placement. Further long-term, randomized clinical trials are needed to give scientific evidence on the benefits of immediate implants over delayed implant placement.

** Key words:**Immediate implants, fresh socket, dental implants, gap, jumping distance, implant stability.

## Introduction

Nowadays advances in clinical techniques and biomaterials have facilitated a great expansion in the indications for dental implant treatment options.

Teeth replacement using dental implants has proven to be a successful and predictable treatment procedure; different placement and loading protocols have evolved from the first protocols in order to achieve quicker and easier surgical treatment times. Immediate placement of a dental implant in an extraction socket was initially described more than 30 years ago by Schulte and Heimke in 1976 (1).

Reductions in the number of surgical interventions, a shorter treatment time, an ideal three dimensional implant positioning, the presumptive preservation of alveolar bone at the side of the tooth extraction and soft tissue aesthetics have been claimed as the potential advantages of this treatment approach (2).

On the other hand, the morphology of the side, the presence of periapical pathology, the absence of keratinized tissue, thin tissue biotype and lack of complete soft tissue closure over the extraction socket have been reported to adversely affect in immediately placed implants (2).

The first classification described the timing of implant placement as mature, recent, delayed or immediate depending on soft tissue healing and predictability of Guided Bone Regeneration (GBR) procedures, however further classifications based on hard and soft tissue healing and treatment time approach were subsequently described, as shown in (Table  Table 1) (3,4).

**Table 1:**
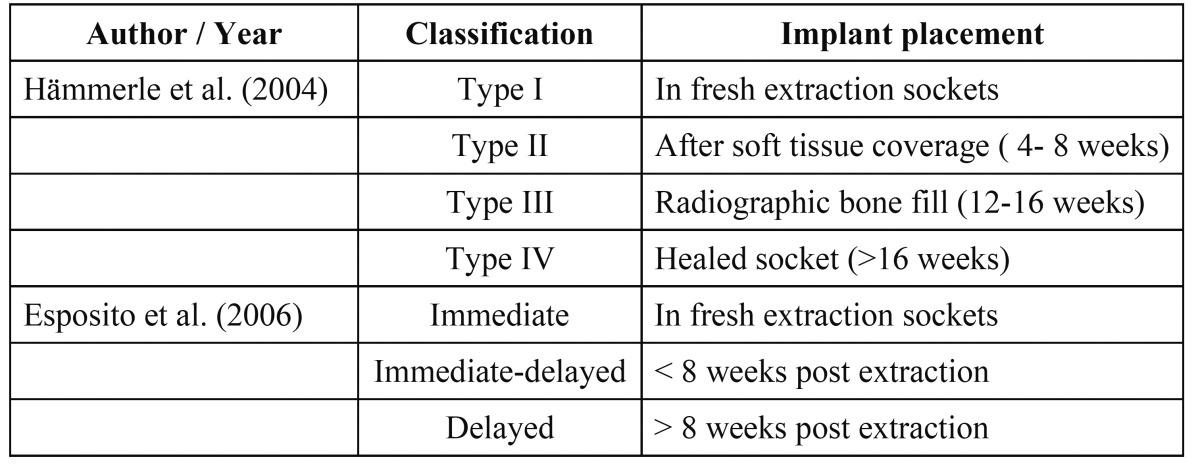
Timing of implant placement.

The efficacy of GBR therapy employing autogenous and non-autogenous particulate materials combined with various membranes to regenerate alveolar bone at the time of tooth extraction has also been demonstrated. Concomitant placement of regenerative materials has been shown to result in predictable, high levels of osseointegration (5).

This study will focus on the review of the current literature on immediate implant placement, in order to understand extraction wound healing and crestal bone loss and the treatment of the jumping distance, as well as several treatment features that affect biological bone and soft tissue response compared to the delayed placement protocol.

The purpose of this review is to answer the following questions:

- Are there significant differences in crestal bone resorption between immediate and delayed implants? Where?

- Do immediate implants have a significant effect on soft tissue recession outcomes?

- Does the presence of periapical infection have an effect on the immediate implant success or survival rate?

- Does the gap treatment minimize crestal bone loss?

- Are there any significant differences in implant stability between immediate and delayed implants?

## Material and Methods

A well-focused question is a very significant step to guide a high- quality and clinically purposeful systematic review. The participant, intervention, comparison, outcome (PICO) approach has been developed to state the objectives and inclusion criteria into a clear structured question (6):

Participants: Patients who needed immediate placement and restoration following extraction of a single tooth.

Intervention: Immediate implant in different clinical situations; upper jaw, lower jaw, anterior or posterior sites, implants with or without guided bone regeneration, and with or without periapical pathology.

Comparison: Immediate implants with or without guided bone regeneration, and immediate implants versus delayed implants.

Outcome: Immediate implant survival and success rates, position of the mucosal margin, mean distance from buccal bone to lingual bone, marginal bone resorption, bone loss, and implant stability.

Search Strategy & Study Selection:

The MEDLINE (PubMed) and The Cochrane Library databases were searched for articles published from 2004 to November 2009. The search was also restricted to articles published in English. The following search terms were used in different combina-tions: immediate implants, extraction socket, fresh socket, dental implants, single implant, gap, jumping distance, implant stabili-ty.

Thirty abstracts were finally selected from 135 titles in the initial search, and the full texts were obtained. Based on the evidence categories of the North of England Evidence Based Guideline Development Project (1996), only randomized clinical trials and prospective clinical trials were included in this review. Therefore, 10 articles were also excluded owing to the reasons shown in (Table  Table 2,  Table 3).

**Table 2:**
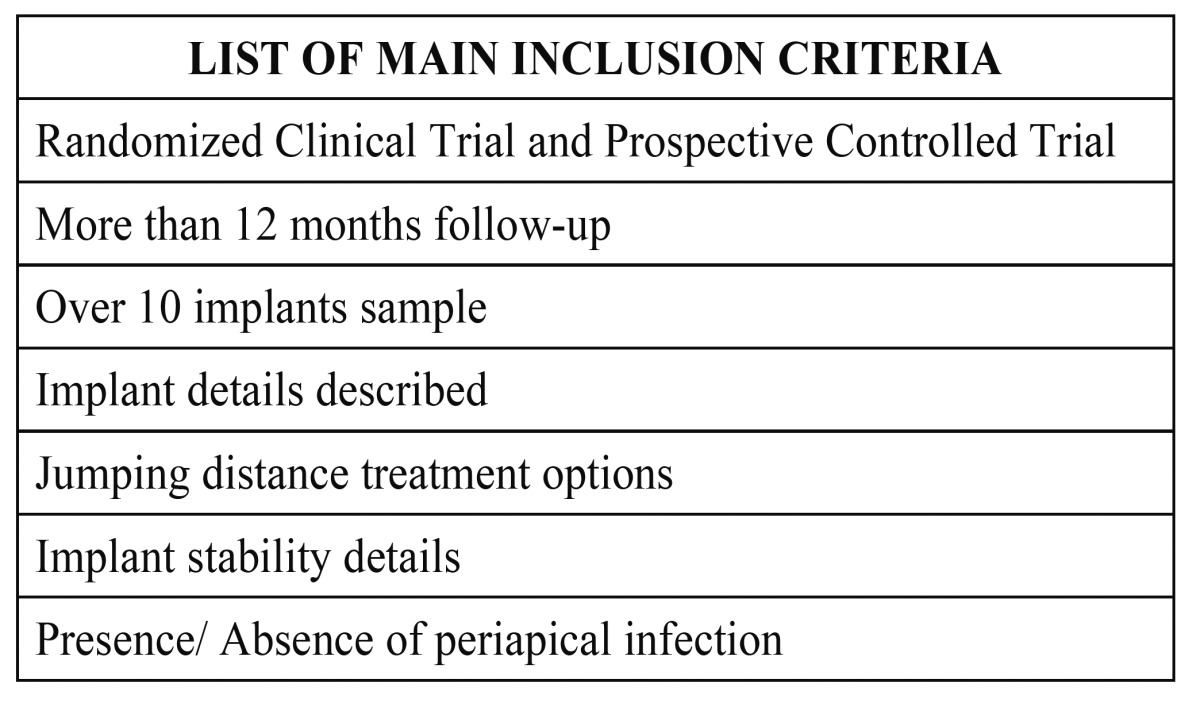
Main inclusion criteria.

**Table 3:**
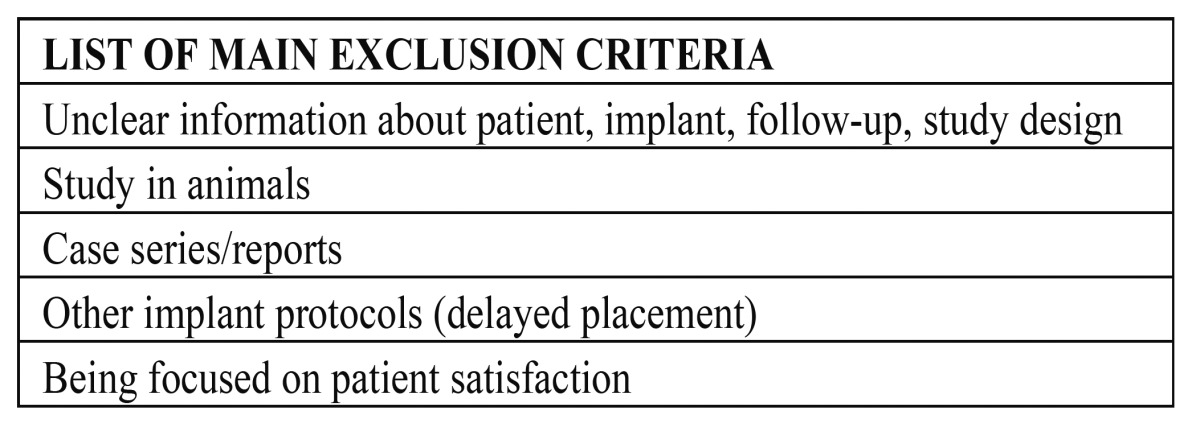
Main exclusion criteria.

The articles finally selected were published in the following dental journals: International Journal of Oral and Maxillofacial Im-plants, Clinical Oral Implants Research, Journal of Oral and Maxillofacial Surgery, Implant Dentistry, Oral Surgery Oral Medicine Oral Pathology Oral Radiology and Endodontology, International Journal of Periodontics and Restorative Dentistry, Clinical Implant Dentistry and Related Research, and Journal of Clinical Periodontology, and were compared with similar reviews from further journals.

The review included studies of patients who needed tooth extraction due to periapical infections, unrestorable caries, trauma or periodontal disease.

When applicable, success rates found in articles were defined according to the criteria described by Albrektsson et al., which in-cludes a reported absence of mobility, pain, peri-implant infection and suppuration, as well as radiographic peri-implant bone loss of ρmm during the first year, followed by 0.2mm for each successive year (7). Implants were classified as surviving if the pu-blished results indicated that the implants were still in function at the time of evaluation, without fulfilling all the success criteria.

Data Extraction:

The following data were obtained using specially designed data extraction search:

Author, year of publication, journal, study design, country of origin, follow-up period, implant characteristics, presence/absence of infection, number of implants in each group, type of implants, use of regenerative procedures in the immediate placement group, crestal bone loss, soft tissue recession, implant stability, implant survival and success rates.

## Results

**Figure 1:**
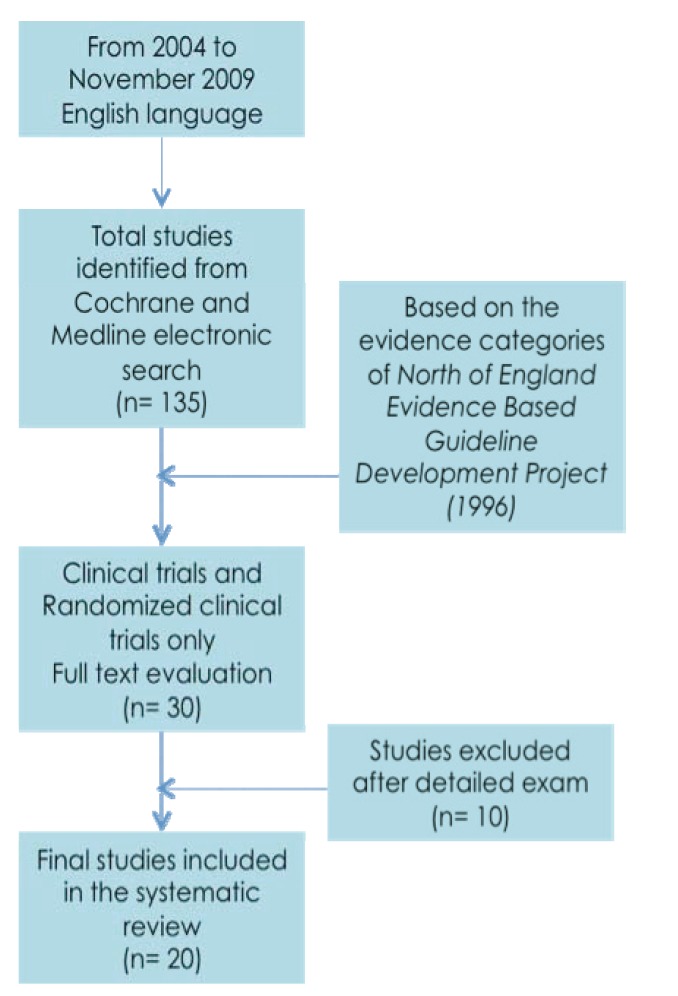
Flow diagram for the search strategy.

From the initial search, 135 citations were found (Fig. 1). Based on the evidence categories of articles, 30 articles were finally selected and full texts were obtained.

Ten studies were excluded after deep analysis (Table  Table 4) (8-17). Four studies were excluded owing to an insufficient follow-up period (less than 12 months) (8,11-13). Another one did not report any specific outcome and focused only on patient satisfaction (17). Two were clinical reports and were not included (9,15) and two more were excluded due to insufficient sample size (no more than 10 implants) (10,14). One study was excluded because implants were placed following a delayed protocol (16).

**Table 4:**
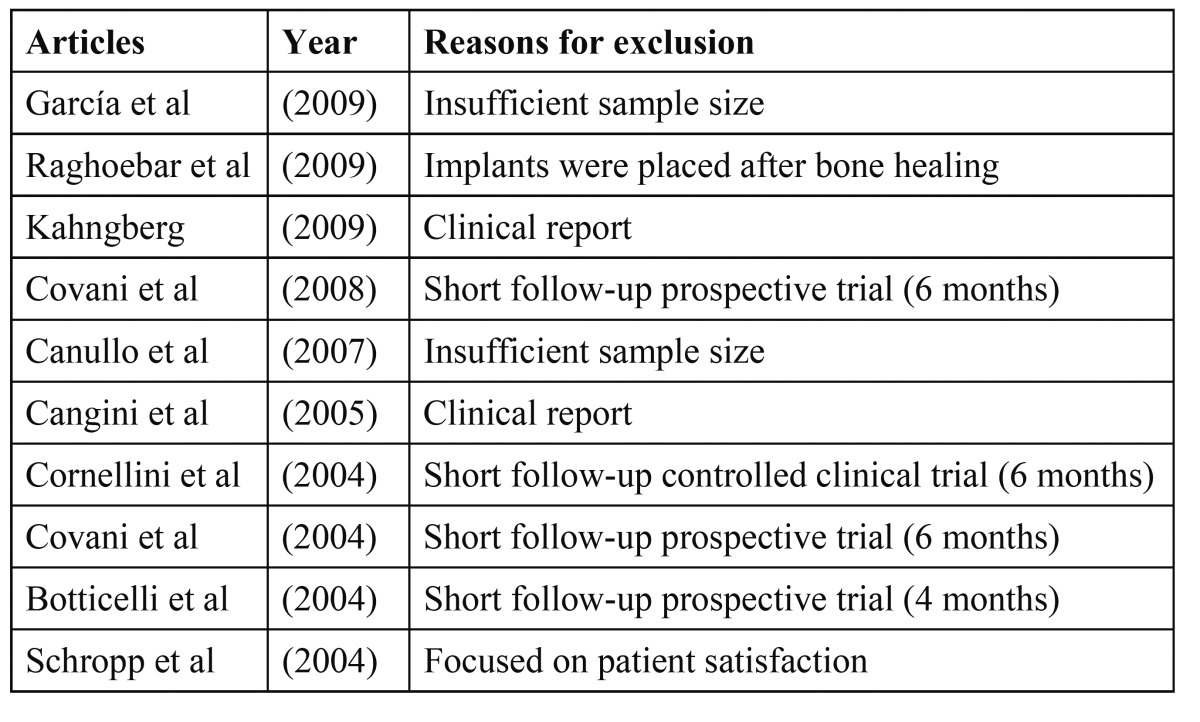
List of excluded articles after the final selection.

Out of the included articles (Table  Table 5a,  Table 5b,  Table 5c), 8 did not include a control group (18-25). Among the included studies, all of them presented a survival rate over 90%. 1139 Immediate implants placed on 904 patients were carefully analyzed with a follow-up of 12 to 60 months.

**Table 5a:**
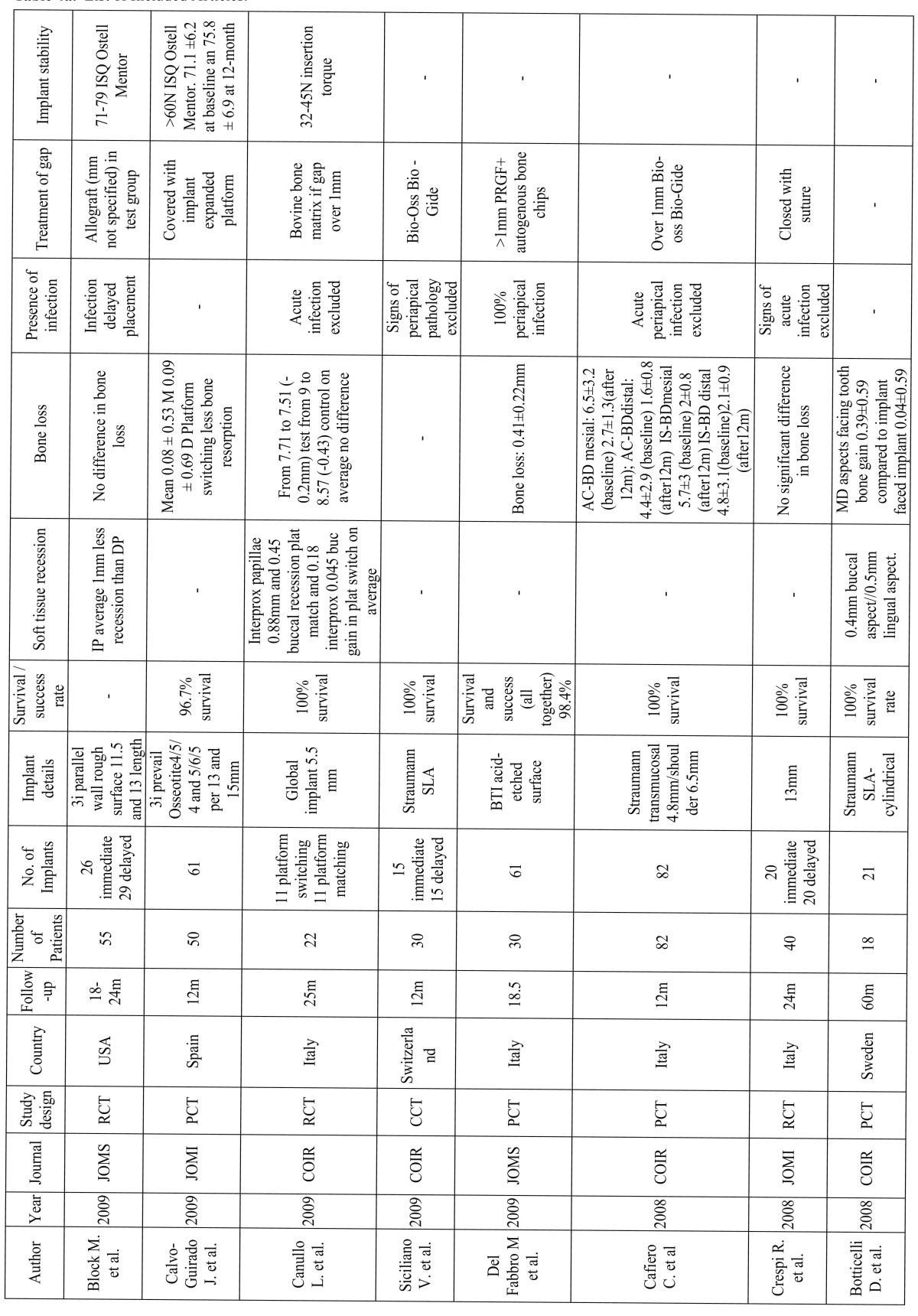
a. List of Included Articles.

**Table 5b:**
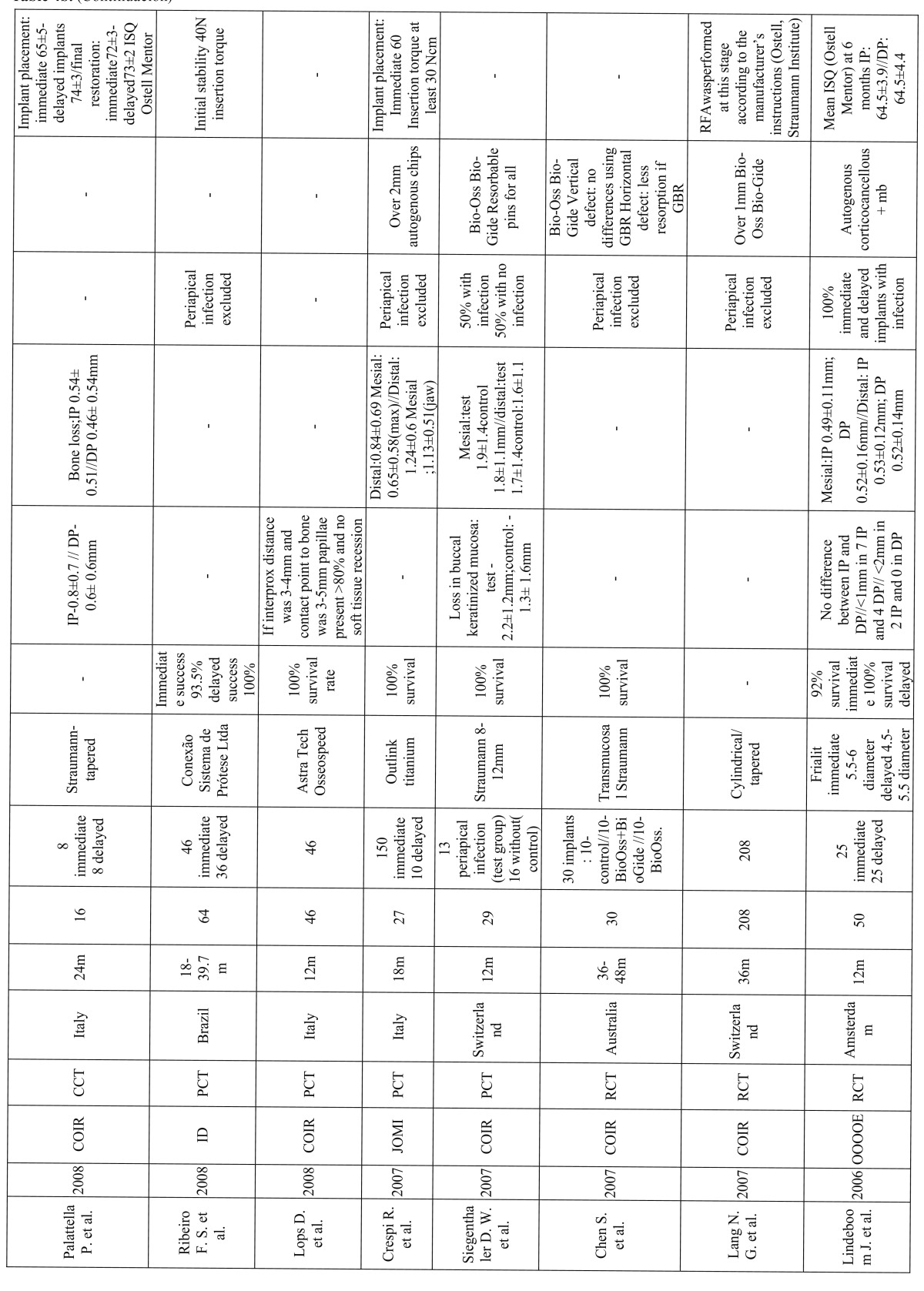
b. (continue table table 5a).

**Table 5c:**
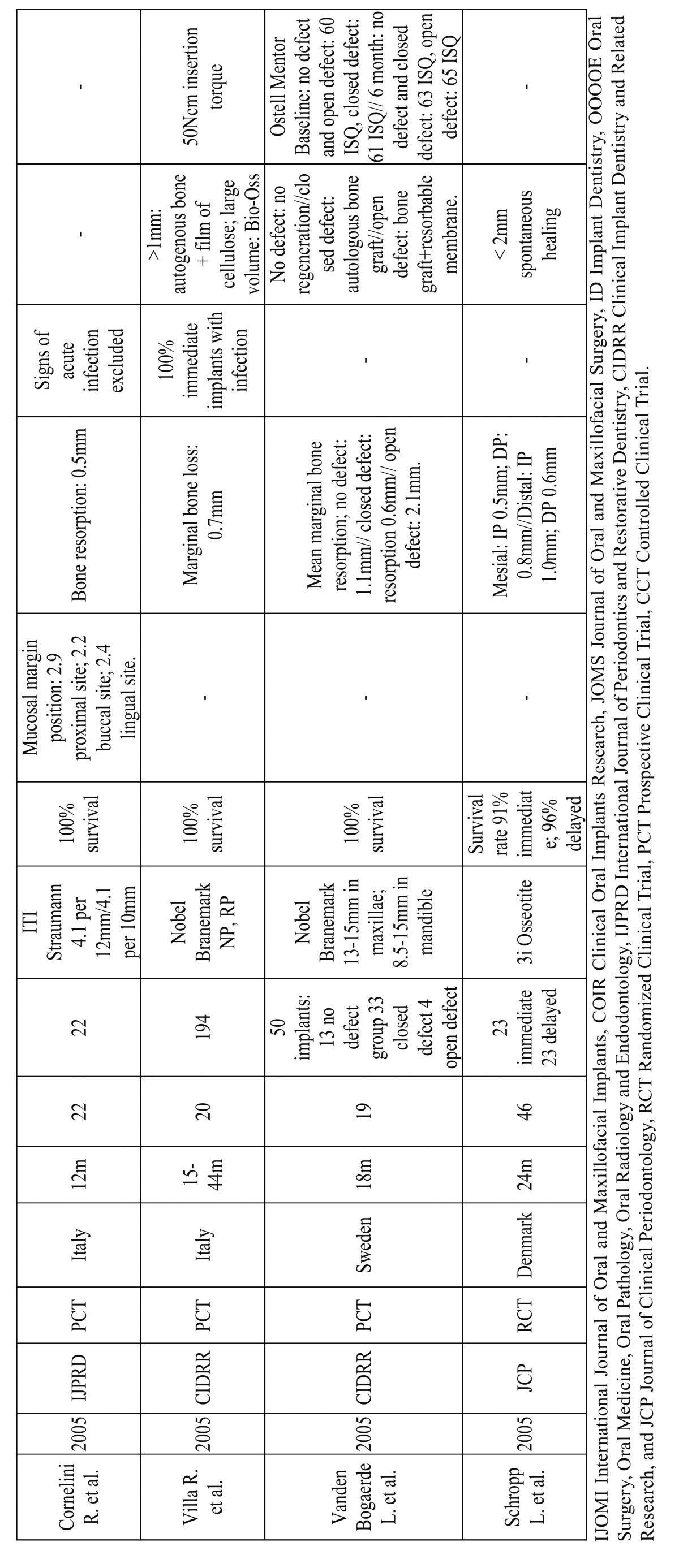
c. (continue table table 5b).

The questions proposed in this systematic review were then answered and compared with the included articles:

Are there significant differences in crestal bone resorption between immediate and delayed implants? Where?

In the prospective clinical study using platform switching, immediate implants showed reduced crestal bone loss (mean 0.08mm ± 0.53 mesial 0.09 ± 0.69 distal) (20). Nevertheless, a similar study found no difference between platform switching and platform matching (on average from 7.71 to 7.51 –0.2mm- vs. 9 to 8.57 -0.4mm-) (26).

A randomized clinical trial of 40 implants were placed in 40 patients in the anterior maxilla–20 immediate implants and 20 dela-yed implants. After a 24-month follow-up period, the control group resulted in a mean bone loss of 1.16mm and the test group of 1.02mm. Once again, there were no statistically significant differences (27).

The survival rate of early-loaded implants placed in fresh extraction sockets of teeth with endodontic and periodontal lesions in the mandible demonstrated no implants failures; a mean marginal bone loss of 0.7mm was recorded during the follow up period. No signs of infection around the implants were detected at any control visit (25).

Differences between delayed-immediate (Im) and the delayed (De) protocols for implant placement were also evaluated. A statis-tically significant radiographic marginal bone resorption had occurred in the Im group (mesial 0.5mm distal 1mm; mean=0.8mm) and in the De group (mesial 0.8mm distal 0.6mm; mean=0.7mm) during the follow-up period. It was demonstrated that probing pocket depths and marginal bone levels after 18 months of loading of the implant-retained crowns were not affected by the pre-sence of peri-implant bone defects immediately after implant placement (28).

Botticelli et al. treated 18 patients with 21 immediate implants. The follow-up period was 5 years. The crestal bone loss was mea-sured in the interproximal areas (m-d aspects facing tooth surfaces showed a higher degree of radiographic bone gain 0.39mm ± 0.59 compared to implant sites that faced adjacent implants 0.04mm ± 0.59) showing a stable bone level over time, even a gain in immediate implants (18).

According to the literature reviewed, measurements of interproximal bone levels are well recorded but few of them show diffe-rences between immediate and delayed protocols.

Do immediate implants have a significant effect on soft tissue recession outcomes?

A randomized clinical trial comparing delayed and immediate implant placement and concluded that immediate placement had an improved average gingival recession outcome of 1mm. However, crestal bone levels were not better preserved with the delayed protocol, and they concluded there were no statistically significant differences (29).

The platform switching study showed an interproximal soft tissue (papillae) of 0.88mm and a 0.45mm buccal recession in plat-form matching and 0.18mm and 0.045mm respectively in platform switching on average; therefore a soft tissue gain was demonstrated (26).

Measurements of soft tissue recessions in a 5-year study with an outcome of 5 buccal sites exhibited soft tissue recession; four of them were positioned in the lower jaw showing the metal margin of the restoration. The mean width of the keratinized mucosa decreased 0.3 mm (buccal site) and 0.4mm (lingual site) during the follow up period (18).

In a 2-year follow-up study on which 16 patients were treated for single tooth replacement and randomly divided into two groups, the test group patients received immediate implants and the control group received implants in healed sites. The following para-meters were evaluated: marginal bone resorption (IP 0.54mm ± 0.51mm vs. DP 0.46mm± 0.54mm) and the position of the mucosal margin (IP: 0.8mm± 0.7mm; DP: 0.6mm± 0.6mm). No statistically significant differences were found between the test and control groups, as in the study performed by Lindeboom et al. 2006 (<1mm in 7 IP and 4 DP; <2mm in 2 IP and 0 in DP) (30).

However, once again, literature of long-term follow-up studies does not show significant differences between both groups.

Does the presence of periapical infection have an effect on the immediate implant success or survival rate?

In most of the studies (19,21,26,27,29,31-35) analyzed in this review, when a periapical infection was present the implant was not placed immediately, instead a delayed placement protocol was performed or patients were just excluded. In fact, in most texts the presence of periapical infection was an exclusion criteria.

However, the clinical outcome of implants immediately placed into fresh extraction sockets of teeth affected by chronic lesions was examined. 17 Patients with periapical infection and 17 patients without it for immediate placement were chosen in another. When infection was present, granulation tissue was removed previously and antibiotics were given (Amoxicillin 750mg 1h before the treatment and 750mg every 8hours, 5 days post-operation). From the initial 34 patients, 4 test and 1 control were excluded due to the lack of primary stability. The rest of the implants presented a survival rate of 100% after 1 year follow-up period. Periapical pathology did not show an increased rate of failures. There was a statistically significant loss of vertical bone height at the adjacent teeth and the implant site, and of buccal keratinized mucosa between baseline and 12 months in both groups (36).

Also, a randomized clinical trial conducted to evaluate clinical outcome when all implants were placed in sockets affected by chronic periapical pathology. 25 Implants were immediately placed (IP) after extraction and 25 after a 3-month healing period (DP, delayed placement). Antibiotic was only given one hour before the surgical procedure (Clyndamicine 600mg). Degranulation of the socket was immediately performed after dental extraction. Gingival recession was more prominent and clinically significant in the IP group (see (Table 5a, 5b, 5c). 2 Implants from the IP group were lost, achieving a survival rate of 92% for IP implants versus 100% for DP implants (23).

Does the gap treatment minimize crestal bone loss?

Bovine bone matrix and collagen membrane is the most common grafting material when the distance between the implant and the bone wall needs to be filled in order to preserve crestal bone (19,25,26,31,33,35,36). Bio-Oss an Bio-Gide were used when the gap exceded 1mm (19,26,33).

Nonetheless, mineralized bone allograft when needed without specifying the gap size or PRGF and autogenous bone graft to cover deshiscences were also used (22,29). Implant macrodesign as an expanded platform was taken in advantage to cover the coronal area of the gap (20). 150 Immediate implants were also placed and if the marginal defect between the implant surface and the inner wall of the extraction socket exceeded 2mm autogenous bone chips were used (32).

A prospective study evaluating healing of marginal defects in immediate transmucosal implants grafted with bovine bone matrix was performed. 30 Implants in the esthetic zone were analyzed of 30 patients that randomly received Bio-Oss (n=10), Bio-Oss and resorbable collagen membrane (n=10) or no graft as a control group (n=10). No significant differences were found regarding vertical defects, although horizontal resorption was significantly greater in the control group (31).

50 Implants were placed in fresh extraction sockets in maxillae and posterior mandibles, including defects around the implants in 19 patients. Temporary prostheses were connected immediately after surgery or within 7 days. Thirteen did not require any type of regenerative procedure (no defect), 33 were filled with autogenous bone (closed defect), and 4 were filled with autogenous bone and also had a resorbable membrane (open defect). After 18 months, none of the implants had failed. In the no defect group, the mean resorption was 1.1mm; in the closed defect group, 0.6mm and in the open defect group 2.1mm (37).

Are there any significant differences in implant stability between immediate and delayed implants?

An important clinical factor to ensure osseointegration is primary implant stability. There are several methods described to mea-sure this parameter. The most common are: during the implant placement with the insertion torque, and resonance frequency analysis (RFA) with the Ostell Mentor device (20,23,29,30,33,37).

In Calvo-Guirado et al. study, immediately placed implants were included with an initial primary stability over 60 ISQ as measu-red with the Ostell Mentor. The mean ISQ values (±SD –standard deviation-) were 71.1 ±6.2 at baseline and 75.8 ± 6.9 at 12-month follow-up. The differences in these results were not statistically significant (20).

Lang et al. compared primary stability of immediately placed implants of tapered versus cylindrical design using RFA. No statis-tically significant differences were found. However, the authors reported that this “study had not been powered to reveal potential differences between standard cylindrical and tapered devices” (33).

## Discussion

This review was designed to provide a broad perspective on the most important aspects of immediate implant placement. Due to data heterogeneity, it was impossible to perform a meta-analysis nor provide recommendations based on conclusive scientific evidence, given the lack of long-term randomized studies and relatively small sample sizes. A preferable technique could not be suggested.

Over time, clinical experience has provided the criteria for immediate implant treatment success: atraumatic tooth extraction, sterilization and minimal invasive surgical approach, as well as implant primary stability (26,34-37).

Quirynen et al. (38) focused their review on immediate versus delayed implant placement. Most papers contained only data on implant loss, but did not provide useful information on implant failure or hard and soft tissue changes. Their data match the results of the present review, in which most of the articles reported data on implant survival rates but not on implant success rates, according to the criteria described by Albrektsson et al. (7).

Moreover, in The Fourth ITI Consensus Conference (November 2009), the advantages and drawbacks of the various points in time for implant placement after tooth extraction were reported. They concluded that immediate implant placement is a more difficult technique than delayed implant placement to allow initial stability and a good prosthetic position. There is also an in-creased risk of mucosal recession. Nonetheless, based on the aesthetic index, 80% of immediate implant sites show satisfactory outcomes. The survival rates of post-extraction implants are high and comparable to those of implants placed in healing sites, like many authors in the present review (39).

Despite many articles previously described limited marginal bone level or gain in immediate implant therapy, caution is needed because few of these studies report radiographic outcomes (4). In contrast, in our review most of the studies analyzed reported the exact millimetres immediate and delayed implants lost during the osseointegration period.

Several reviews reported that the immediate implant treatment using autogenous bone grafts or xenografts may improve the process of bone formation between the implant and the surrounding socket walls as well as survival rates (2,5). They observed that several studies have suggested that small gaps between implants and extraction sockets would fill with bone grafting procedures or without them. These data are in accordance with some results obtained in the present review (10,19,22,23,25,26,29,31-33,35).

With regard to the gap between the socket wall and the implant, it was reported that if 

the jumping distance is over 2mm, grafting is recommended. Smaller distances could heal spontaneously (2,5,40). In our review, similar results on grafting the jumping distance have been contrasted. However, there is a current controversy as to which is the best grafting material (autograft, xenograft or allograft), and how big the gap should be (1-2mm).

In the Clinical Outcomes of ITI consensus, one extensive review provided strong evidence that immediate placement does not prevent vertical or horizontal resorption of the ridges in post-extraction sites. Bone augmentation following immediate placement reduces horizontal resorption on the facial bone. However, these augmentation procedures appear not to influence vertical resorption on the facial bone (39). The review also provided strong evidence that augmentation procedures are more successful with immediate implant placement than with delayed implant placement.

Few studies comparing implant stability between delayed and immediately placed implants seem to be available in the literature. From the reviewed studies, it seems that ISQ values are somewhat lower in immediately placed implants compared to implants placed in pristine bone (30). However, these differences tend to disappear overtime (23,30). ISQ values seem to increase progres-sively during healing over the first few months in immediate implants (20,23,30). Further controlled clinical studies should be performed in order to verify these findings.

## Conclusions

There is not enough reliable evidence proving higher success of immediate implant placement over delayed placement. Post-extraction implants have survival rates similar to implants placed on healed sites. Nevertheless, some guidelines could be extracted from this review’s data:

- Interproximal bone level and soft tissue recession.

Crestal bone as well as soft tissue preservation could be achieved with either by immediate implant placement following tooth extraction or by a delayed protocol. No statistically significant differences were found despite the review of medium and long term follow-up studies.

- Treatment of the gap between implant and bone wall

There is no consensus whether bone augmentation with GBR at immediate implants placed into fresh extraction sites are necessary, and which is the most predictable procedure. However Bio-Oss and membranes therapy seem to show a higher position of the gingival margin.

- Presence of periapical infection

Chronic periapical infection is a risk factor but not an absolute contraindication for immediate implant placement. However, debridement of the alveolus should be made. The presence of a periapical infection should be carefully weighed.

- Primary implant stability

Primary implant stability is an important factor in achieving osseointegration. Several methods have been used to quantify this parameter, such as insertion torque values and resonance frequency analysis (RFA). However, few scientific studies reveal com-parative data between immediate and delayed implant placement. It seems that there are no significant differences between primary stability of immediate and delayed implants, but in both cases implant stability increases during the healing process.

Based on this review of the literature tackled, immediate implant placement following tooth extraction might be a viable alterna-tive to delayed placement. However, it requires a careful case selection and a specific treatment protocol because it is a very sensitive technique and more difficult to execute than a conventional protocol.
